# Effect of plate design on maintenance of anterior and posterior gaps and posterior tibial slope after cyclic loading in medial open‐wedge high tibial osteotomy: A biomechanical study using porcine's tibia

**DOI:** 10.1002/jeo2.12036

**Published:** 2024-06-19

**Authors:** Yoshiya Nibe, Tsuneari Takahashi, Tomohiro Matsumura, Tatsuya Kubo, Katsushi Takeshita

**Affiliations:** ^1^ Department of Orthopaedic Surgery, School of Medicine Jichi Medical University Shimotsuke Japan; ^2^ Department of Orthopaedic Surgery Ishibashi General Hospital Shimotsuke Japan; ^3^ Department of Emergency and Critical Care Medicine Jichi Medical University Shimotsuke Japan

**Keywords:** anteromedially plate design, biomechanical study, high tibial osteotomy, porcine tibia, posterior tibial slope

## Abstract

**Purpose:**

We aimed to investigate whether a plate adapted to the anatomy of the proximal medial porcine's tibia would provide maintenance of the anterior gap (AG), posterior gap (PG) and posterior tibial slope (PTS).

**Methods:**

Twenty‐seven porcine tibias were biomechanically evaluated by performing MOWHTO and placing TOMOFIX (*n* = 9), AC plate (*n* = 9) and TriS (*n* = 9) anteromedially. Cyclic testing (800 N, 2000 cycles, 0.5 Hz) was performed to investigate the PTS over time for MOWHTO. The particular displacement calculated from the maximum to the minimum point with the load‐displacement curve along the mechanical axis during cyclic testing, the final AG and PG changes at the osteotomy site, the increased PTS calculated by subtracting AG from PG after 2000 cycles were compared among the three groups. The displacement was evaluated by repeated‐measures analysis of variance (ANOVA), and changes in AG and PG and increased PTS were evaluated by one‐way ANOVA. The sample size for *α* and *β* errors were <0.05 and <0.20, and the effect size was 0.64 for one‐way ANOVA and 0.49 for repeated‐measures ANOVA.

**Results:**

There were no significant differences in displacement among the groups. A significant difference was observed in the AG (*p* = 0.044) and PG (*p* = 0.0085) changes. There were no significant differences in increased PTS among the groups.

**Conclusion:**

When anteromedially placed, the AC plate and TriS resulted in significant maintenance of AG and PG compared with that of TOMOFIX in MOWHTO after cyclic loading.

**Level of Evidence:**

Level Ⅳ.

**Abbreviations**
ACL: anterior cruciate ligament
AG: anterior gap
ANOVA: analysis of variance
MOWHTO: medial open wedge high tibial osteotomy
PG: posterior gap
PTS: posterior tibial slope

## INTRODUCTION

**Figure 1:**
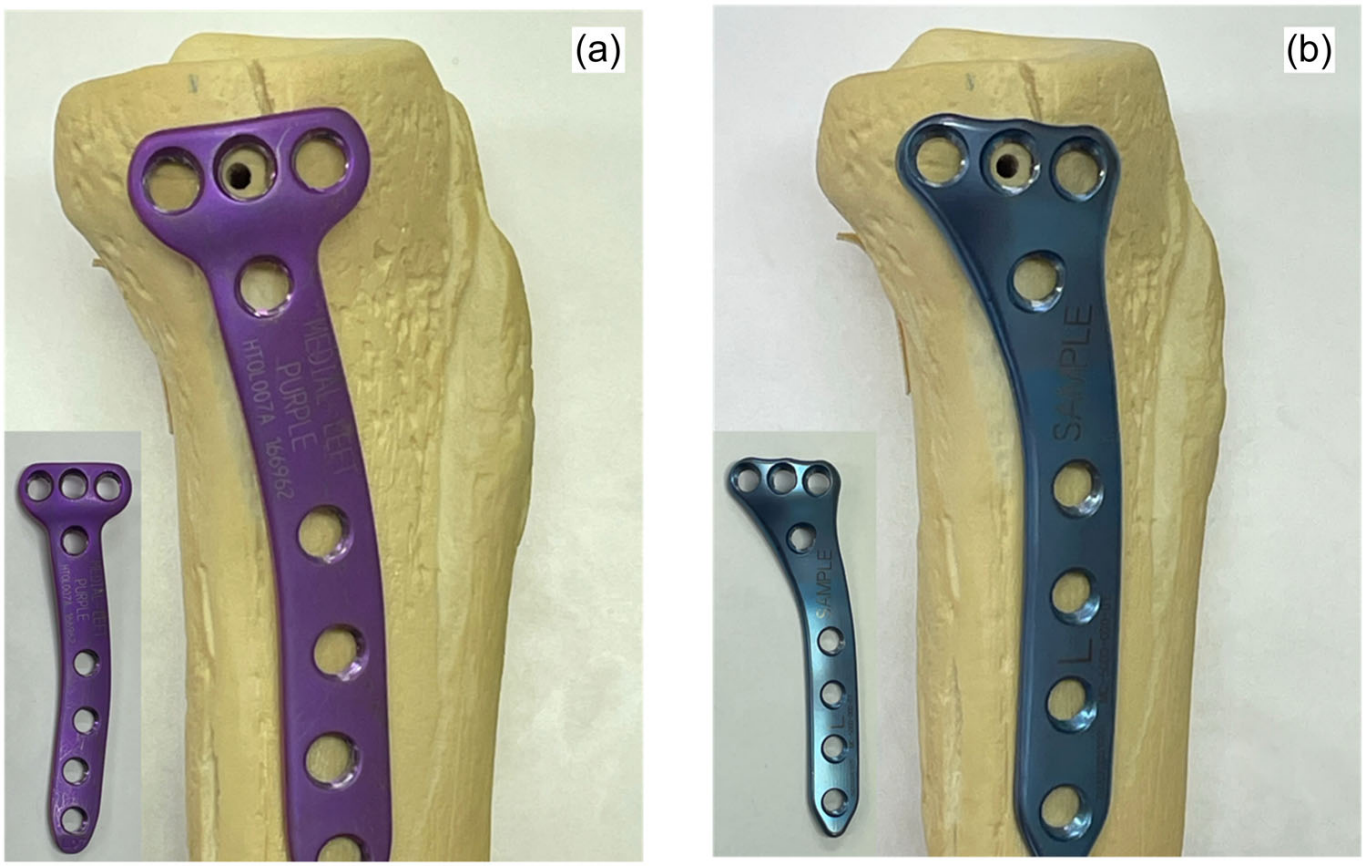
(a) TriS (Olympus Terumo Biomaterials). (b) AC plate (Omic). These plates allow the insertion of screws from the medial side at the proximal tibial articular surface and from the anteromedial side at the diaphysis.

Currently, medial open‐wedge high tibial osteotomy (MOWHTO) is the widely performed knee osteotomy procedure for treating varus knee osteoarthritis [19]. However, increased posterior tibial slope (PTS) over time has recently been reported [4, 5, 16], resulting in anterior cruciate ligament (ACL) insufficiency [9, 18]. Some previous studies have investigated the influence of lateral cortical hinge axis orientation on PTS [6, 14]. To solve this problem, more rigid fixation using a locking plate is recommended. Previous studies comparing several plates have reported that implants adapted to the tibial anatomy provide more stable fixation and mechanical stability [3, 11]. However, no study has focused on whether the plate design for MOWHTO influences the degree of PTS after cyclic loading. In Japan, TriS (Olympus Terumo Biomaterials) plates are distributed and widely used (Figure 1a). Recently, AC plates (Omic) with different screw directions on the proximal and distal sides, similar to that of TriS plates, have also become available (Figure 1b). Relatively, these plates can be positioned medially near the articular surface and anteromedially at the proximal of the tibia to conform to the tibial anatomy. Therefore, we aimed to investigate whether a plate adapted to the anatomy of the proximal medial tibia would provide maintenance of anterior gaps (AG), posterior gaps (PG) and PTS. We hypothesized that the TriS and AC plates would provide maintenance of AG, PG and PTS compared with the anterointernal TOMOFIX (DePuySynthes) when anteromedially placed.

## MATERIALS AND METHODS

### Study design

**Figure 2:**
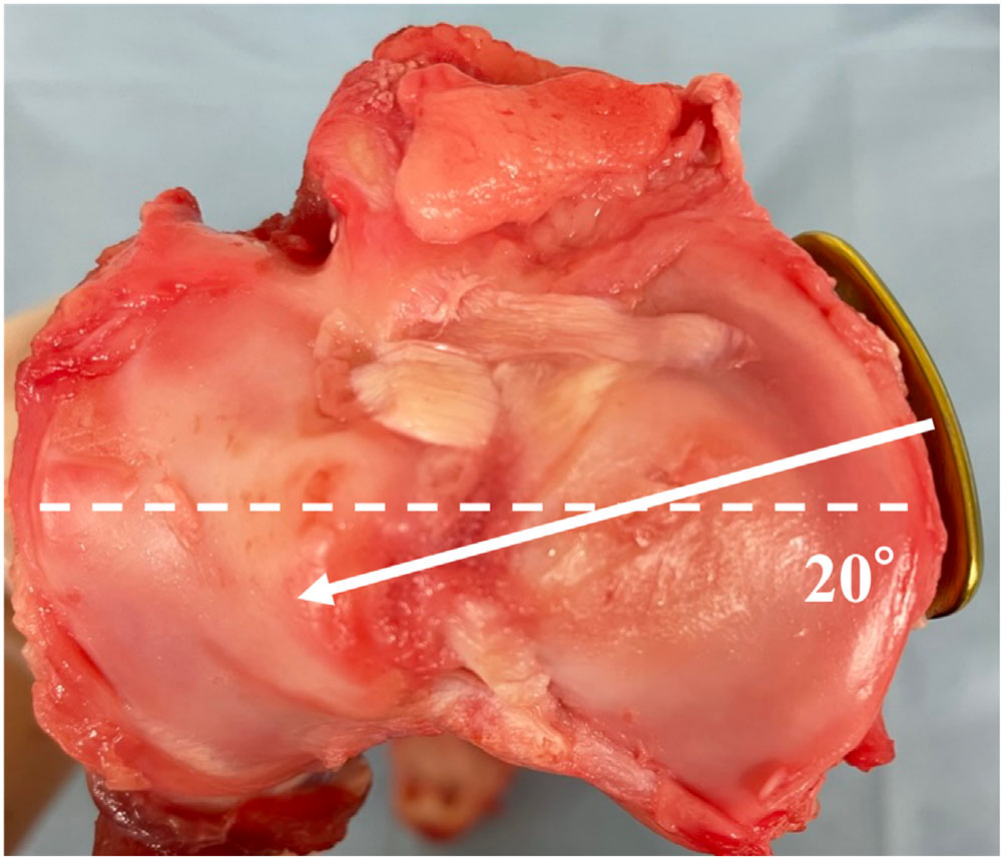
Anteromedial plate positioning (right knee). The screw direction (white arrow) is 20° from the transverse diameter of the tibial plateau (dotted line).

Animal experiments were performed in a biomechanics laboratory of our institution in accordance with the regulations of the Institution's Animal Care and Use Committee. Obtaining ethical approval from this committee was waived due to the ex vivo nature of this study. Twenty‐seven fresh porcine knees (age: 6 months; weight range: 180–200 kg, Tokyo Shibaura Zouki) were used and divided into three groups. TOMOFIX (*n* = 9), AC plate (*n* = 9) and TriS (*n* = 9) were used in the first, second and third groups, respectively. The plate position was fixed to the medial tibia with a thread direction of the tibial diaphysis at 20° (anteromedial position) from the transverse diameter of the tibial plateau, according to Takeuchi et al. [23]. (Figure 2). The TriS and AC plates are placed anteromedially and designed to allow the proximal screws to be inserted transversely and parallel to the proximal tibial articular surface. The specimens were thawed for at least 24 h before use. The medial proximal tibial angle (MTPA) of specimens before MOWHTO was not excessively valgus or varus macroscopically.

### Surgical procedure

MOWHTO with a 6 mm opening wedge was performed on all specimens according to standard surgical instructions [22]. First, a 1.6 mm K‐wire was punctured 45 mm from the medial joint line and directed towards the fibular tip. To obtain directional confirmation, the K‐wire was punctured through the opposite cortex, just touching the fibular tip. Then, an oscillating saw was used. Osteotomy was performed along the K‐wire direction from the posterior to the anterior medial tibial cortex. Osteotomy was stopped 5 mm from the lateral tibial cortex, leaving a hinge for opening. We performed an additional osteotomy of the anterior third of the tibial cortex, including the tibial tuberosity fragment, in the anterior forehead plane at an angle of 110° to the axial plane. The osteotomy was carefully opened with three chisels while applying a bulging gas force to the distal end. A 6 mm gap was identified and the osteotomy line was kept parallel. The TOMOFIX (group T), AC plate (group A) and TriS (group S) were placed medial to the proximal tibia and plate fixation was performed. The proximal and distal bone fragments were fixed with four locking monocortical and four bicortical screws, respectively. Among the three groups, the plates were fixed to the bone in the same manner. All screws were fixed using a sleeve guide to ensure that they were placed in the same direction. In all groups, no artificial bone was inserted into the open wedge.

### Biomechanical evaluation

**Figure 3:**
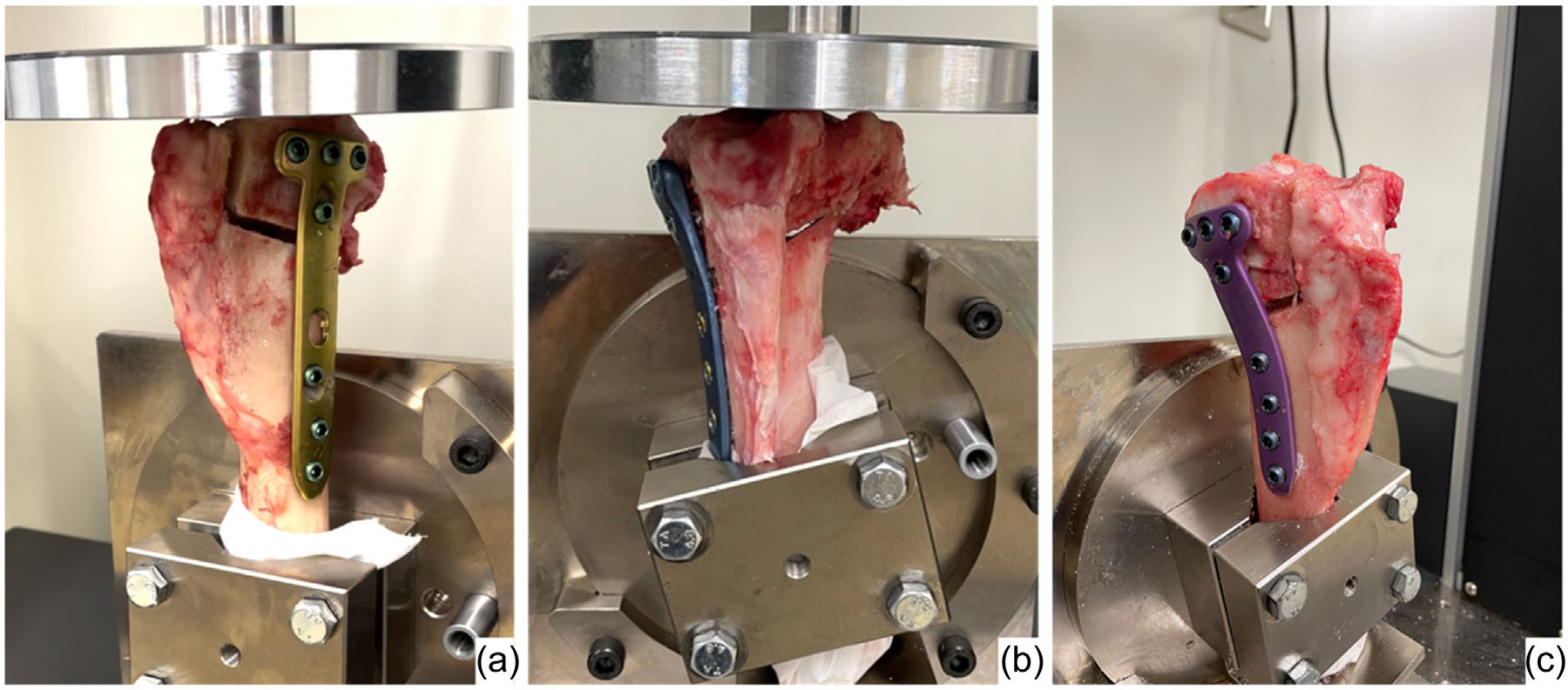
Specimen preparation. (a) Specimen after MOWHTO with TOMOFIX applied to the right knee. (b) Specimen after MOWHTO with AC plate applied to the left knee. (c) Specimen after MOWHTO with TriS applied to the left knee. MOWHTO, medial open wedge high tibial osteotomy.

**Figure 4:**
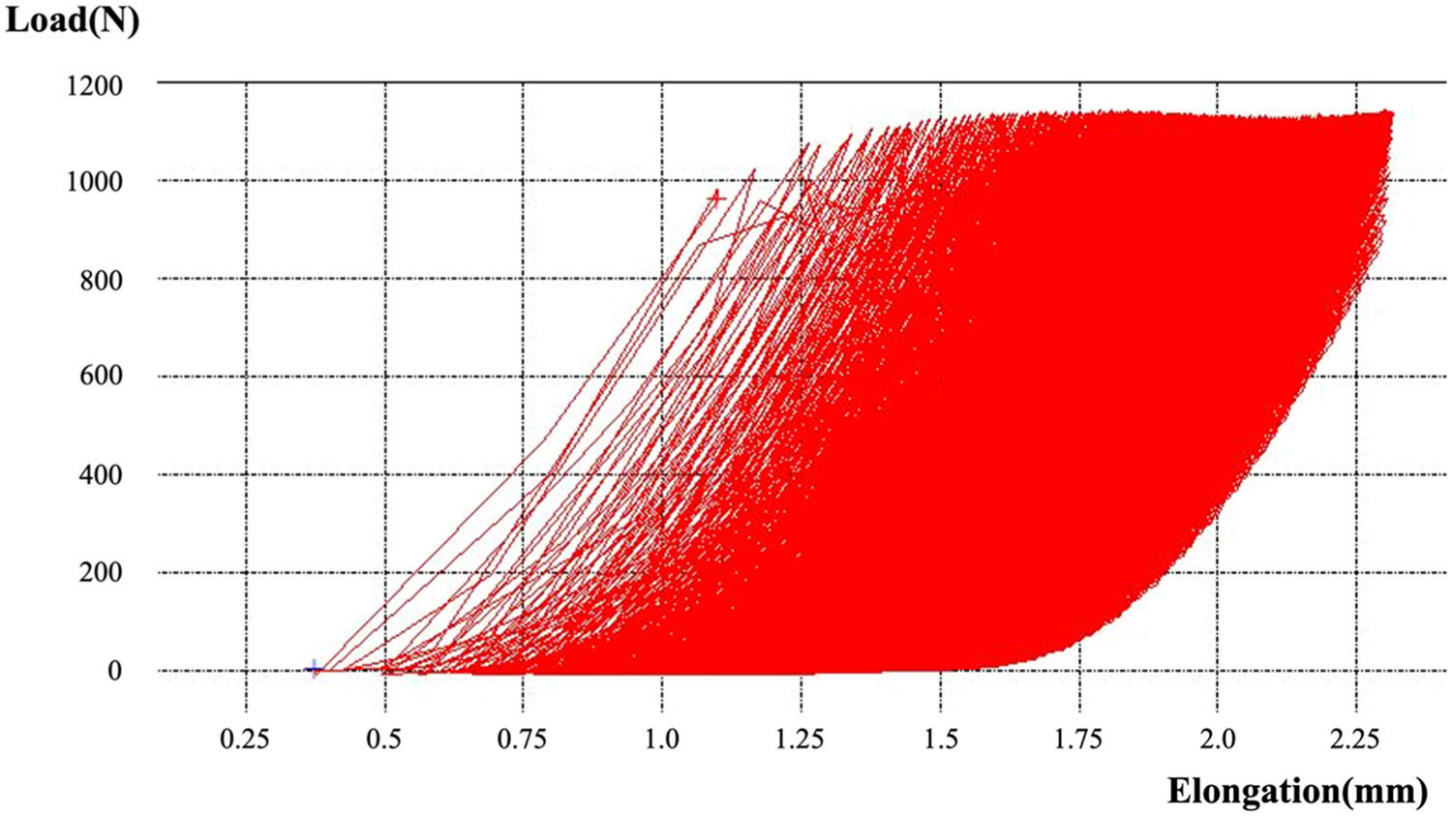
Schematic image of the load‐displacement curve generated during cyclic testing with this software.

Cyclic testing was performed to investigate the translation patterns of the three fixation constructs. This was done along the postoperative mechanical axis of the tibia using a tensile testing machine (Tensilon RTG 1310, Orientec Co. Ltd.) equipped with a specially designed set of grips. This measurement system was similar to that used in a previous biomechanical study [1, 13, 17]. After cutting 10 cm of the distal tibia, 4 cm of the distal portion of each tibia was clamped using a custom‐made jig (Figure 3). MTPA after MOWHTO was about 90° and the proximal tibial articular after setting in testing machine was level with the plane of loading macroscopically. Load‐displacement curves were generated using specific software (Tensilon Advanced Controller for Testing, Orientec Co.) (Figure 4). Then, cyclic loading of up to 800 N was applied to the tibia (2000 cycles, 0.5 Hz). During cyclic loading, the PTS of specimens moved with increasing posterior tilt as the load increased and decreasing posterior tilt as the load decreased. Load conditions were determined according to Takeuchi et al.'s previous study [23]. The alignment of specimens after cyclic loading was not excessively altered macroscopically. The actuator displacement at the 10th, 100th, 500th, 1000th, 1500th and 2000th cycles was calculated and determined using the software. The AG and PG changes in the osteotomy site were measured using a precision caliper with an accuracy of 0.1 mm. Measurements of the AG and PG changes were taken by two researchers and averaged over three measurements. Therefore, intra‐ and interrater reliability was improved. Increased PTS was calculated by subtracting AG from PG and using the anteroposterior diameter of the proximal tibial articular surface (average 50 mm). The anteroposterior diameter of the porcine's proximal tibial articular was measured at an average of 50 mm; therefore, the anteroposterior diameter was calculated at 50 mm in this study.

### Statistical analyses

A repeated‐measures analysis of variance (ANOVA) with Bonferroni post‐hoc analysis was used to evaluate the displacement among the three groups. To evaluate the AG and PG changes and increased PTS among the three groups, we performed a one‐way ANOVA with Turkey post‐hoc analysis. All data are presented as mean ± standard deviations. *p* Values < 0.05 were considered statistically significant. A priori power analysis was performed using G* Power 3.1 (Franz Paul, Kiel) [7]. The sample sizes for *α* and *β* errors were <0.05 and <0.20, and the effect size was 0.49 for repeated‐measures ANOVA and 0.64 for one‐way ANOVA. All statistical analyses were performed using the EZR software [8]. The minimum sample size by one‐way ANOVA of AG and PG changes and increased PTS was 66, and the minimum sample size by repeated‐measures ANOVA of actuator displacement was 291. In this study with a smaller sample size than the minimum sample size, a difference in each test could be interpreted as a large difference. Hence, as the effect size of repeated‐measures ANOVA and one‐way ANOVA were larger than Cohen's effect size guideline, any difference in each group would indicate highly significant differences.

## RESULTS

### Displacement during cyclic loading

**Figure 5:**
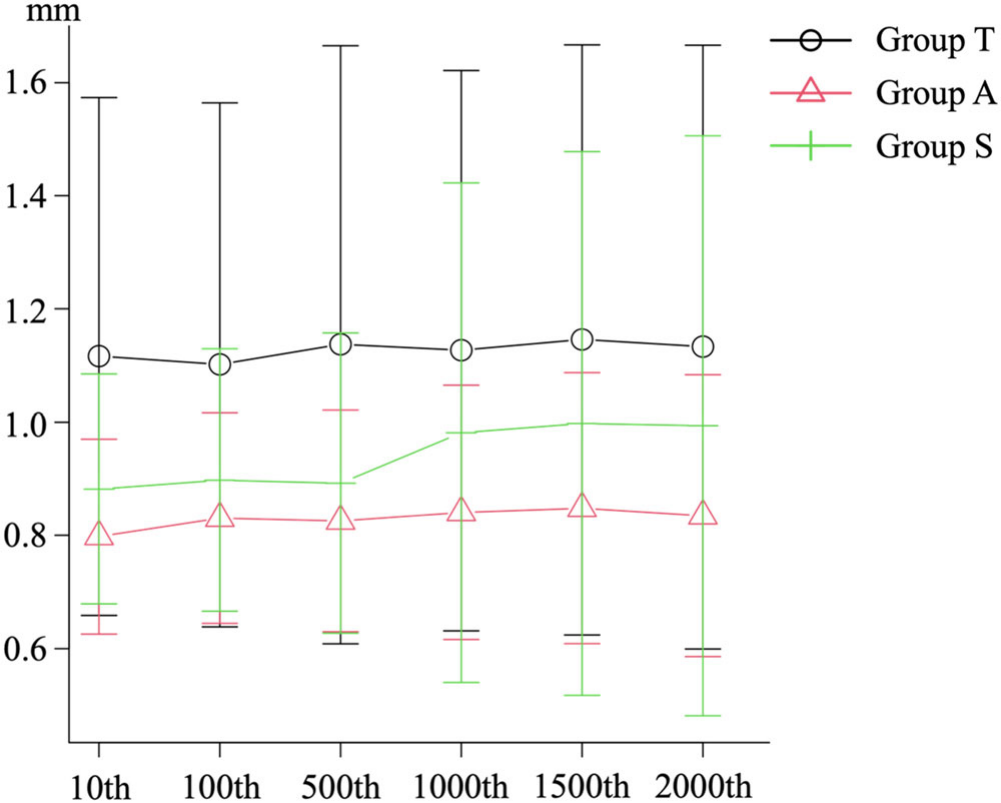
Displacement among the three groups.

There were no significant differences in the displacement at the 10th, 100th, 500th, 1000th, 1500th and 2000th cycles (Table 1) (Figure 5).

**Table 1:** The displacement among the three groups.

Parameters	Group T (*n* = 9)	Group A (*n* = 9)	Group S (*n* = 9)
10th (mm)^a^	1.12 (0.43)	0.80 (0.16)	0.88 (0.19)
100th (mm)^a^	1.10 (0.44)	0.83 (0.17)	0.90 (0.22)
500th (mm)^a^	1.14 (0.50)	0.83 (0.18)	0.89 (0.25)
1000th (mm)^a^	1.13 (0.47)	0.84 (0.21)	0.98 (0.42)
1500th (mm)^a^	1.15 (0.49)	0.85 (0.23)	1.00 (0.45)
2000th (mm)^a^	1.13 (0.50)	0.83 (0.23)	0.99 (0.48)

^a^ Data are expressed as mean (standard deviation).

### Changes in the AG and PG

**Figure 6:**
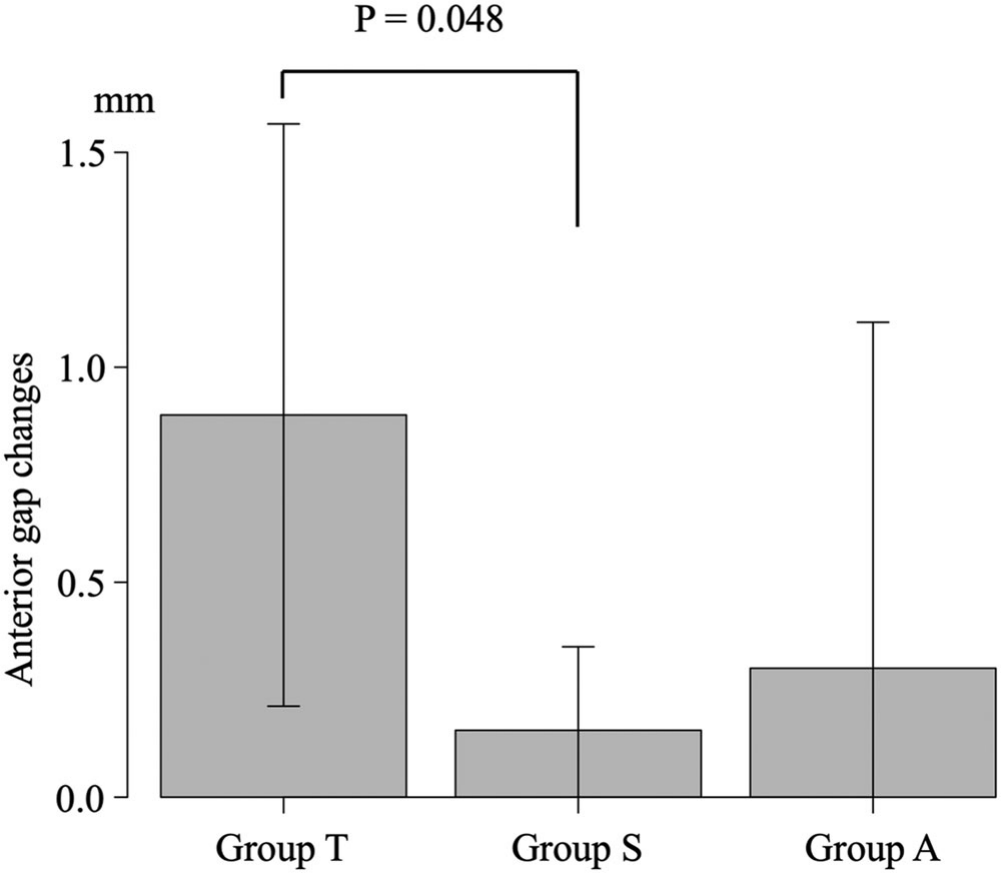
The anterior gap changes among the three groups.

**Figure 7:**
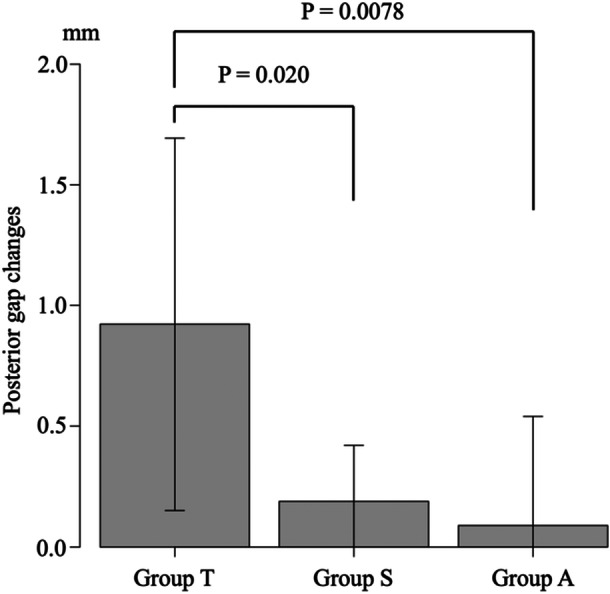
The posterior gap changes among the three groups.

There were significant differences in the AG changes among the three groups (*p* = 0.044) (Table 2) and post‐hoc analysis revealed significant differences between TriS and TOMOFIX (*p* = 0.048) (Figure 6). There were significant differences in the PG changes among the three groups (*p* = 0.0053) (Table 2). Post‐hoc analysis revealed a significant difference between the AC plate and TOMOFIX (*p* = 0.012) and between TriS and TOMOFIX (*p* = 0.035) (Figure 7).

**Table 2:** The anterior and posterior gap changes and the posterior tibial slope changes among the three groups.

Parameters	Group T (*n* = 9)	Group A (*n* = 9)	Group S (*n* = 9)	*p* Value^b^
The anterior gap changes^a^	0.89 (0.68)	0.30 (0.80)	0.16 (0.19)	0.044
The posterior gap changes^a^	0.92 (0.77)	0.089 (0.45)	0.19 (0.23)	0.0053
The posterior tibial slope changes^a^	0.04 (0.76)	0.24 (0.70)	0.01 (0.39)	0.58

^a^ Data are expressed as mean (standard deviation).

^b^ Comparison among groups by use of one‐way analysis of variance.

### Changes in increased PTS

**Figure 8:**
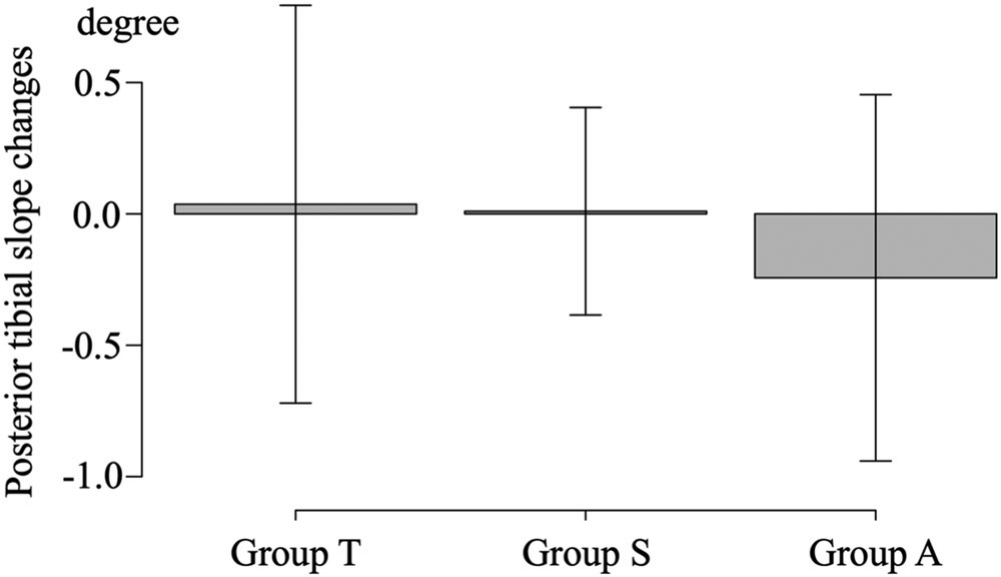
The posterior tibial slope changes among the three groups.

There were no significant differences in increased PTS among the three groups (Table 2) (Figure 8).

## DISCUSSION

In this study, the TriS used in MOWHTO had less AG and PG, and the AC plate used in MOWHTO had less PG after cyclic loading than anteromedially TOMOFIX. To the best of our knowledge, this is the first study that quantitatively clarified that the amount of AG and PG after cyclic loading depends on the plate designs in MOWHTO. The results of our biomechanical evaluation were consistent with our hypothesis.

A previous biomechanical study has reported that the anatomical anteromedially plate of the tibia has stronger fixation as compared with other medial locking plates [3]. In the present study, the TriS and AC plates had a relatively more anatomical design of a tibia than the anteromedially placed TOMOFIX, which may have resulted in a lesser PG in the TriS and AC plate groups. Previously, TOMOFIX was recommended because of its lesser complications than the other plates used in MOWHTO [2, 11]. However, Nakamura et al. reported that an anteromedially placed TOMOFIX has complications of screw breakage and increased PTS [15]. To address these complications, they decided to position the TOMOFIX medially and insert the long screws transversely to the proximal tibial articular surface [15]. The anatomical plate of a tibia has the advantages of good radiographic outcomes and appropriate fixation as compared with TOMOFIX [12, 20, 21]. In the present study, the TriS and AC plates were relatively anatomical plates, which allowed for long screw insertion into the proximal articular of the tibia. Therefore, the AC plate had less change of PG compared with anteromedially TOMOFIX, and TriS had less changes of AG and PG than anteromedially TOMOFIX.

There were no significant differences in increased PTS among the three groups. However, increased PTS was calculated from the changes in the AG and PG. If AG and PG are changes of the same distance, increased PTS remains the same. However, the sinking of proximal medial articular of tibia would be expected to increase varus because of the sinking of the osteotomy. TriS had less change in AG and PG and less increased PTS, which might not only maintain PTS but also reduce varus changes at the coronal plane. Although there were no macroscopically apparent alignment changes in this study, radiological evaluation might have revealed changes. Thus, TriS would exhibit fewer gap changes after cyclic loading compared with other plates.

Adapted plates for the proximal tibia have a lesser gap change than anteromedially TOMOFIX; therefore, it is better to use adapted plates for the proximal tibia while performing MOWHTO. The clinical relevance of this study was that MOWHTO using an adapted plate for the proximal tibia would be effective for maintaining correction until bone was union.

The present study has several limitations. First, a porcine model was used; thus, the present results may not be directly applicable to clinical practice. Furthermore, porcine tibias might have different fixation forces from those of humans. However, it was reported that porcine knees are similar to human knees in many aspects [1, 10, 17]. Second, this study only evaluated the time‐zero structural characteristics using specimens with MOWHTO anteromedial plates. Therefore, the influence of any biological healing response was not considered. Moreover, the actual in vivo loading may not be reflected because we only conducted ex vivo studies on a portion of the tibias. Hence, future in vivo studies are required to determine whether similar results would be obtained. Third, the machine used in this study could only evaluate axial direction. In this study, however, increased PTS can be assessed by measuring the AG and PG. Hence, measuring the AG and PG after cyclic loading could have supplemented the sagittal evaluation. Fourth, there might be slight malalignments because they have not been examined radiologically. As no obvious alignment abnormality occurred, the experiment continued with two researchers, who did not find any issues. Finally, the limit of the machine load was 2000 cycles. If more cycles had been performed, there might have been differences in displacement and increased PTS.

However, despite several limitations, this is the first study to investigate the biomechanical advantage of TriS and AC plates when compared with the anteromedially placed TOMOFIX for MOWHTO using cyclic loading conditions. Our study will serve as the basis for biomechanical studies of the anterior medial plate in vivo.

## CONCLUSIONS

When MOWHTO was performed with an anteromedial plate, the TriS and AC plates resulted in significant maintenance of PG compared with TOMOFIX after cyclic loading. Additionally, TriS resulted in significant maintenance of AG compared with the anteromedially placed TOMOFIX in MOWHTO after cyclic loading.

## AUTHOR CONTRIBUTIONS

Conception and design of this study were performed by Yoshiya Nibe, Tuneari Takahashi and Tatsuya Kubo. Acquisition of data was done by Yoshiya Nibe and Tsuneari Takahashi. Analysis and/or interpretation of data was carried out by Yoshiya Nibe and Tsuneari Takahashi. Drafting of the article was done by Yoshiya Nibe, Tsuneari Takahashi, Hironori Hai, Tomohiro Matsumura and Katsushi Takeshita. All authors have contributed significantly to the study, approved the article and agreed with the submission.

## CONFLICT OF INTEREST STATEMENT

The authors declare no conflict of interest.

## ETHICS STATEMENT

Animal experiments were performed in a biomechanics laboratory of our institution in accordance with the regulations of the Institution's Animal Care and Use Committee. Obtaining ethical approval from this committee was waived due to the ex vivo nature of this study.

## Data Availability

Data and materials of this study are available from the corresponding author upon reasonable request.
